# Mitochondrial RNA editing PPR proteins can tolerate protein tags at E as well as at DYW domain termini

**DOI:** 10.3389/fpls.2014.00127

**Published:** 2014-04-03

**Authors:** Nadja Brehme, Anja Zehrmann, Daniil Verbitskiy, Barbara Härtel, Mizuki Takenaka

**Affiliations:** Molekulare Botanik, Universität UlmUlm, Germany

**Keywords:** RNA editing, plant organelles, PPR protein, E domain, DYW domain

## Introduction

In plastids and mitochondria of plants, RNA editing changes numerous cytidines to uridines. The nucleotides to be edited are selected by *trans*-acting proteins which are structurally characterized as pentatricopeptide repeat (PPR) proteins (Kotera et al., 2005; Schmitz-Linneweber and Small, 2008; Takenaka et al., 2013b). The approximately 35 amino acids long elements each attach to a specific nucleotide moiety and several tandemly arranged elements establish selective contact to a unique RNA sequence pattern. The PPR parameters defining the nucleotide specificity were identified by computational analysis and confirmed by experimental retargeting and by crystal structures (Barkan et al., 2012; Ke et al., 2013; Takenaka et al., 2013a; Yagi et al., 2013; Yin et al., 2013). Half of the PPR family of about 450 proteins in flowering plants consists only of these repeats and few further domains if any. This group of PPR proteins is involved in RNA processing events other than RNA editing (Schmitz-Linneweber and Small, 2008). The about 200 PPR proteins associated with RNA editing are C-terminally extended by the so-called E domains, the function of which is so far unknown. About half of these proteins contain an additional C-terminal domain with key features of deaminases in the form of characteristic amino acids which may bind an essential zinc atom (Hayes et al., 2013). However, so far no deaminase activity has been found, one of these domains rather shows an RNase activity (Nakamura and Sugita, 2008). This domain terminates in most instances with the name giving amino acid triplet DYW. In several such PPR RNA editing factors in plastids and mitochondria, the DYW domain can be deleted without compromising the function of the protein in editing (Okuda et al., 2009), while in others the DYW domain is required (Zehrmann et al., 2010). In some of the E class PPR RNA editing factors, addition of a DYW domain does not affect their competence in editing (Verbitskiy et al., 2012a). So far the functional parameters are unclear which distinguish those protein factors that require the C-terminal DYW extension beyond the E domain from those that do not. The E domain is in all of these RNA editing PPR proteins essential and cannot be removed.

To learn more about the function and structural features of these extension domains, we probed the importance of native C-termini of several PPR proteins in mitochondria by adding an additional protein domain. These chimeric proteins were assayed for their *in vivo* RNA editing competence to complement respective *Arabidopsis thaliana* mutants.

## Most E domain PPR editing proteins are fully functional with a C-terminal GFP extension

**Figure 1:**
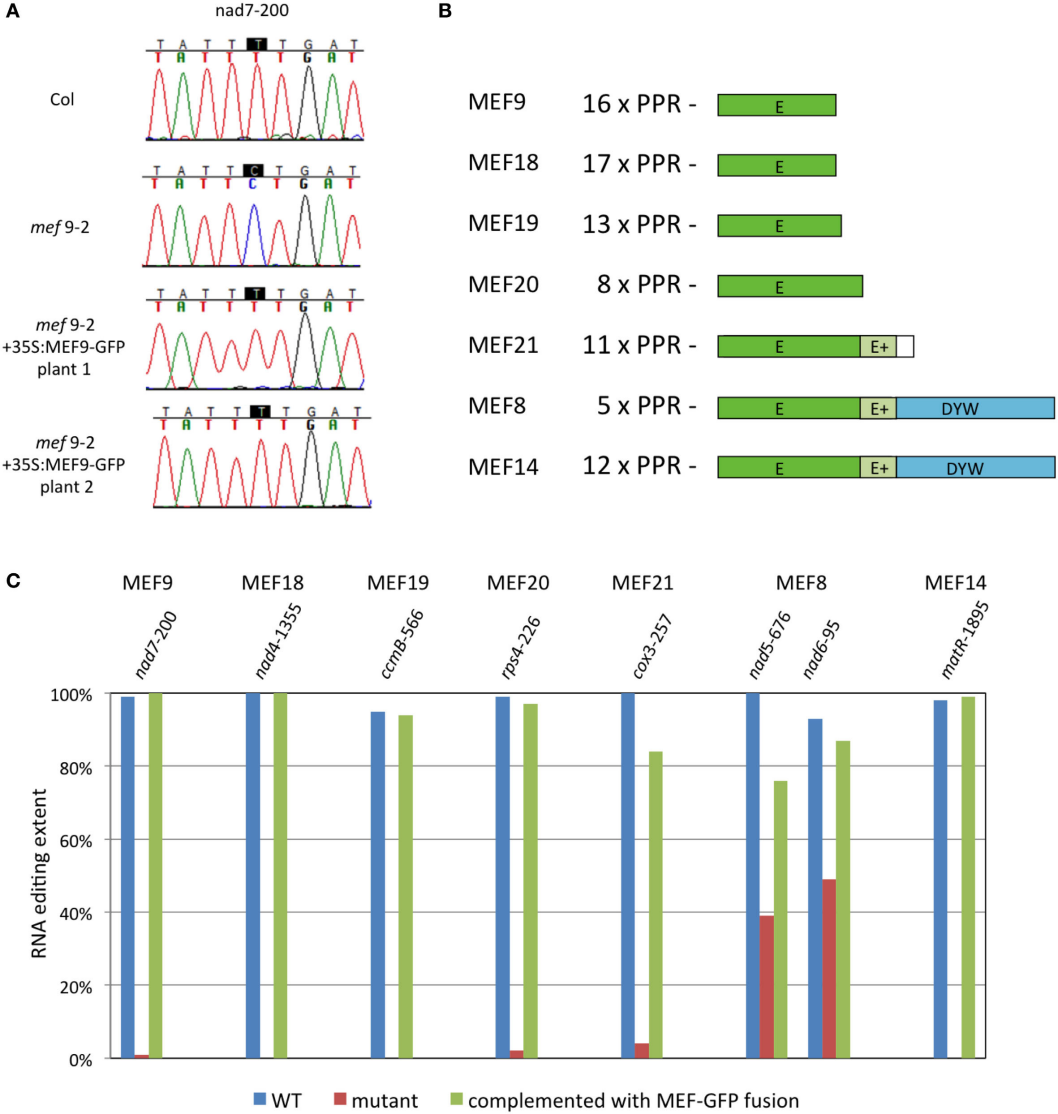
**RNA editing specific PPR-proteins terminating with E- or DYW-domains tolerate an additional protein tag. (A)** Analysis of MEF9 is shown as a representative example. The single target site of MEF9 in the mitochondrial mRNA coding for subunit 7 of the respiratory chain complex I is nearly completely edited to U in wild type *Arabidopsis thaliana* plants of the Columbia ecotype (Col; Takenaka, 2010). In the *mef9-2* mutant plant, RNA editing at nucleotide *nad7*-200 (counted from the A in the AUG translational start codon) is below the background threshold of 10%. The chimeric protein of MEF9 with GFP attached to the C-terminus of the E domain fully recovers editing at this site (Plants 1 and 2). Color traces are: G, black; A, green; T, red; C, blue. **(B)** Structure of the PPR RNA editing proteins analyzed as chimeric MEF-GFP proteins. The number of the PPR elements are given and the approximate lengths of the E domains are depicted. Several proteins contain an additional extension termed E+. The white box in MEF21 indicates an extension of the open reading frame beyond the E/E+ domains but without similarity to the DYW region. **(C)** Bar graph of the complementation levels achieved at respective target sites in mutant plant lines transformed with the corresponding chimeric MEF-GFP proteins. Shown are the maximal editing levels observed in at least 1 of the 11 or 12 transgenic plant lines analyzed for each mutant. *Arabidopsis thaliana* plants were grown, transformed and analyzed as described (Zehrmann et al., 2009; Verbitskiy et al., 2012a). Chimeric gene constructs were cloned under control of the 35S promoter. Individual transformants were selected and propagated independently. RNA editing was verified by cDNA sequence analysis in each transgenic plant line, percentages of editing were calculated by relative peak heights of C and T sequence signals, respectively (Zehrmann et al., 2009).

As representatives for the PPR type site-specific mitochondrial RNA editing factors (MEF) terminating with an E domain, we selected MEF9, MEF18, MEF19, MEF20, and MEF21 (Takenaka, 2010; Takenaka et al., 2010). Their respective open reading frames were extended by adding a C-terminal GFP coding sequence (260 amino acids) in frame and cloned under control of the 35S promoter. The chimeric proteins were analyzed in *Arabidopsis thaliana* plants for their competence to complement respective mutants. Fusion proteins of MEF9, MEF18, MEF19, and MEF20 with GFP all showed full competence in RNA editing at their respective target sites and gave transformants fully recovered to wild type level editing (Figure 1). For MEF9 and MEF19, all twelve, and for MEF18 all 11 regenerated mutant plants complemented with the respective C-terminal GFP fusion protein regained wild type RNA editing levels. The MEF20-GFP chimera complemented fully in 1 of 12 stably transformed plants, more than 90% editing levels were seen in another five plants, and between 50 and 90% editing were recovered in another four plants. Two plants did not show any alteration of the absence of editing. Nine of the MEF21-GFP transformed mutant plants regained the ability for RNA editing which increased from 0 to 21–84%, 2 of 12 plants complemented with MEF21-GFP showed no editing, none of them fully recovered the apparently complete nucleotide conversion of the wild type. The variation of editing recovery between individual transformants can most likely be attributed to varying cosuppression effects and/or the individually differing nuclear integration locus of the transgene, which is known to strongly influence transgene expression levels. In addition, *in vivo* editing levels sustained by introduced PPR proteins have been found to correlate unpredictably with their RNA levels (Okuda et al., 2008) probably due to further posttranslational regulation, wherefore we did not analyze mRNA levels generated off the transgenes.

## DYW domain PPR editing proteins can also tolerate a C-terminally attached GFP

Of the PPR type site-specific mitochondrial RNA editing factors terminating with a DYW domain, we tested MEF14 (Verbitskiy et al., 2011) and the rather short MEF8 protein (Verbitskiy et al., 2012b). With MEF14-GFP fusion proteins, 3 of 12 regenerants recovered the editing level of wild type plants, showing that a GFP can be tolerated at the C-terminus. A further four plants showed 80–90% recovery, up from no discernible editing in the mutant, another four regained 30–80% and one showed no recovery beyond the background of the sequence analysis.

The MEF8 protein contains only five recognizable PPR elements, which would hardly be sufficient for sequence-specificity, yet does affect unique sites when mutated (Verbitskiy et al., 2012b). In *mef8-2* mutant plants, site *nad5*-676 is edited to only about 40% compared to the 100% of the wild type, and site *nad6*-96 is reduced to about 50% from about 95% editing. In 10 of 11 regenerated complemented plants the MEF8-GFP chimera raised the editing levels at both sites by 10–40%, indicating that the recombinant protein is functional in editing.

## Biochemical application of functional E and DYW PPR editing proteins with a C-terminal GFP tag

The PPR type site-specific mitochondrial RNA editing factors are required for editing at one, or at most few, specific sites. It is presently not clear whether some of them are sufficient for editing their target site(s), since so far no successful *in vitro* editing assays with a clean full length PPR protein and its target RNA have been reported. So far, only *in vitro* assays showing the RNA-PPR protein connection (Okuda et al., 2009) and the nuclease activity of a partial PPR protein containing the DYW domain have been published (Nakamura and Sugita, 2008). It is more likely that at least *in vivo* a more complex editosome performs the editing reaction since a number of additional proteins are required for or at least influence RNA editing in either or both plant organelles. Foremost are the MORF proteins whose major function in the cell may be in RNA editing and which can directly contact specific editing PPR proteins (Bentolila et al., 2012; Takenaka et al., 2012). Other RNA binding proteins have been found to influence RNA editing such as ORRM and other RRM binding domain containing organellar proteins (Tillich et al., 2009; Sun et al., 2013). Recently, an enzyme of the tetrapyrrole biosynthesis pathway has been found to be required for RNA editing at selected sites in the chloroplast transcriptome (Zhang et al., 2014).

The here evaluated construction of MEF-GFP fusion proteins from two sub-groups of PPR proteins, those terminating with the E and those with an additional DYW domain, will allow to detect interactions with other proteins. These transgenic mutant plants can be used for pull-down experiments to identify associated proteins. The confirmed functionality of the fusion proteins suggests that the added GFP moieties do not inhibit necessary interactions with other protein molecules. It seems prudent to first establish the functionality of PPR proteins modified by comparatively large tags as the entire GFP protein which may distort the *in vivo* relevance of protein-RNA or protein-protein interactions. The major obstacle hindering biochemical investigations, the very low abundance of the editing PPR proteins, can possibly be circumvented or alleviated by selecting transgenic plant lines with elevated MEF-GFP expression levels.

## The conserved terminal amino acids can be extended in some, but not all DYW PPR editing proteins

The DYW amino acid triplet is generally at the very C-terminus of this domain. However, several PPR mitochondrial RNA editing factors show variations of the DYW triplet to other triplets such as DFW in MEF7, DSW in OTP86, or EYW in MEF8S. Altogether, about a third of the mitochondrial and plastid “DYW” editing factors contain triplets other than DYW. Nevertheless, the C-terminal amino acid triplet being conserved in so many proteins implies a functional constraint. Similarly, some of the His and Cys residues implicated in the potential deaminase activity (Salone et al., 2007) are not found in all DYW domains. However, these variants may be explained if not all DYW domains are actually functional as suggested by the distinct requirement for this domain in individual PPR proteins.

In this context, the MEF11 mutant *mef11-2* shows that in this PPR protein, the DYW domain is essential for some targets, but not for all (Verbitskiy et al., 2010). The T-DNA insertion removes half of the domain, the DYW triplet and 56 upstream amino acids, but leaves the amino acid pattern of the potential deaminase intact. Surprisingly, deletion and complementation assays showed that this entire domain is not required and can be removed from the MEF11 protein (Zehrmann et al., 2011). Conversely, the DYW domain is essential in the MEF1 protein, truncated versions ending with the E domain cannot recover editing in the respective mutant. Concomitantly, the MEF1 protein does not tolerate a C-terminal extension by a His-tag, suggesting that some essential DYW termini have to be free and are not functional when extended (Zehrmann et al., 2010). The here observed partial complementation of the tagged MEF8 protein may also indicate that masking of the C-terminus by the GFP moiety does inhibit the activity and function of this DYW PPR protein. Similar differential results for several plastid editing PPR proteins support the inference that the DYW is essential in only some PPR proteins. The likely explanation is that the DYW function can be substituted by another protein containing this region. Prominent support for this surmise comes from the instance of the DYW1 and the CRR4 proteins, these proteins can be present in trans or can be linked into a single amino acid chain to edit a plastid site (Boussardon et al., 2012). By analogy, absent or non-functional DYW domains will be supplied by additional PPR proteins interacting with the site specific PPR protein possibly as a direct heteromer or with the support of one or more of the other factors contributing to the editosome complex. Evidence for direct dimer formation of PPR proteins has come from crystallization studies, in which the PPR10 protein forms a dimer, with or without bound target RNA (Ke et al., 2013; Yin et al., 2013).

## Perspective

In plant organellar RNA editing, specific sites are addressed by PPR proteins which all contain C-terminal extension domains. We here report that in *Arabidopsis thaliana*, several PPR editing proteins terminating with an E or with an additional DYW domain tolerate the addition of a GFP moiety. Since the DYW motif marks the C-terminus of the respective domain, it is surprising that a GFP protein can be added in frame to some DYW proteins without disturbing their apparent functionality.

### Conflict of interest statement

The authors declare that the research was conducted in the absence of any commercial or financial relationships that could be construed as a potential conflict of interest.
